# Dynamic monitoring of G_i/o_-protein-mediated decreases of intracellular cAMP by FRET-based Epac sensors

**DOI:** 10.1007/s00424-017-1975-1

**Published:** 2017-04-06

**Authors:** Ursula Storch, Julie Straub, Serap Erdogmus, Thomas Gudermann, Michael Mederos y Schnitzler

**Affiliations:** 1 Walther Straub Institute of Pharmacology and Toxicology, Ludwig Maximilians University of Munich, Goethestr. 33, 80336 Munich, Germany; 2 DZHK (German Centre for Cardiovascular Research), Munich Heart Alliance, Munich, Germany; 3 Comprehensive Pneumology Center Munich (CPC-M), German Center for Lung Research, Munich, Germany

**Keywords:** cAMP, FRET, Epac sensor, G_i/o_-coupled receptors, G_s_-coupled receptors

## Abstract

Analysis of G-protein-coupled receptor (GPCR) signaling, in particular of the second messenger cAMP that is tightly controlled by G_s_- and G_i/o_-proteins, is a central issue in biomedical research. The classical biochemical method to monitor increases in intracellular cAMP concentrations consists of a radioactive multicellular assay, which is well established, highly sensitive, and reproducible, but precludes continuous spatial and temporal assessment of cAMP levels in single living cells. For this purpose, Förster resonance energy transfer (FRET)-based Epac cAMP sensors are well suitable. So far, the latter sensors have been employed to monitor G_s_-induced cAMP increases and it has remained elusive whether Epac sensors can reliably detect decreased intracellular cAMP levels as well. In this study, we systematically optimize experimental strategies employing FRET-based cAMP sensors to monitor G_i/o_-mediated cAMP reductions. FRET experiments with adrenergic α_2A_ or μ opioid receptors and a set of different Epac sensors allowed for time-resolved, valid, and reliable detection of cAMP level decreases upon G_i/o_-coupled receptor activation in single living cells, and this effect can be reversed by selective receptor antagonists. Moreover, pre-treatment with forskolin or 3-isobutyl-1-methylxanthine (IBMX) to artificially increase basal cAMP levels was not required to monitor G_i/o_-coupled receptor activation. Thus, using FRET-based cAMP sensors is of major advantage when compared to classical biochemical and multi-cellular assays.

## Introduction

G-protein-coupled receptors (GPCRs) constitute a large family of membrane proteins that transduce extracellular signals into cellular responses by activating intracellular signal transduction pathways. GPCRs are commonly activated by a plethora of small molecules and hormones, and some GPCRs can even perceive physical and chemical cues such as mechanical forces (summarized in [28]), voltage [2, 3, 22, 23], or ions [29]. Since GPCRs are involved in many physiological and pathophysiological processes and represent molecular targets for about 30% of all approved drugs [20], a detailed analysis of GPCRs and their signaling pathways is of utmost importance and may be leveraged to further improve medical treatment. The two best characterized families of effector enzymes regulated by GPCRs are phospholipases C (PLC) and adenylyl cyclases (ACs). Several members of the former enzyme family are activated by G_q/11_-proteins. Intracellular cAMP levels can either be elevated as a consequence of G_s_-protein-dependent activation of ACs or decreased following engagement of G_i/o_-protein-coupled receptors resulting in inhibition of AC [12]. Thus, the second messenger cAMP is tightly regulated by G_s_- and G_i/o_-coupled receptors.

**Fig. 1:**
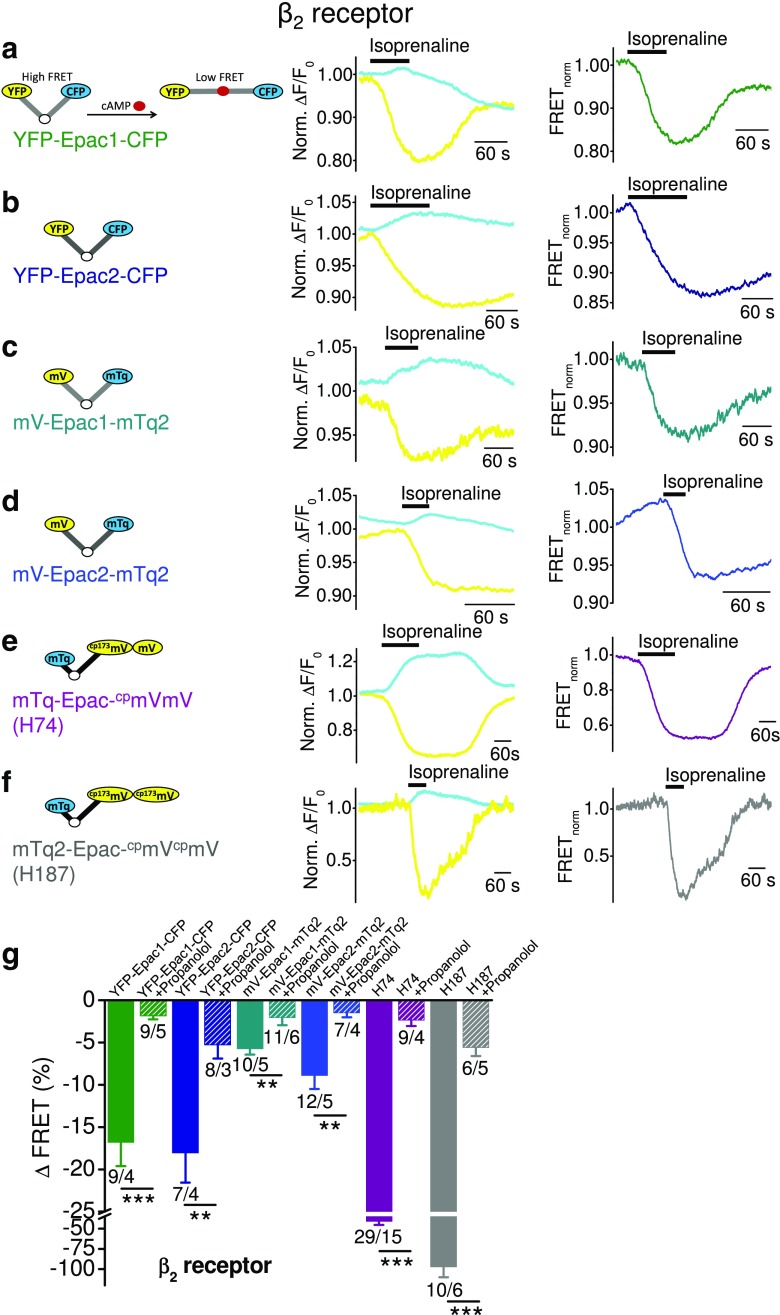
Optimized FRET-based cAMP sensors are effective in monitoring G_s_-mediated cAMP level increases. FRET measurements with HEK293 cells endogenously expressing G_s_-protein-coupled β_2_ receptors together with one of the indicated FRET-based cAMP sensors: YFP-Epac1-CFP (**a**), YFP-Epac2-CFP (**b**), mV-Epac1-mTq2 (**c**), mV-Epac2-mTq2 (**d**), mTq-Epac-^cp^mVmV (H74) (**e**), and mTq2-Epac-^cp^mV^cp^mV (**f**). **a**–**f** Representative FRET measurements are displayed showing time courses of the normalized yellow and cyan fluorescence signals (*left*) and of the normalized FRET signals (*right*). Black bars indicate application of the β receptor agonist isoprenaline (200 μM). *Left insets*, schematic illustration of the different FRET-based cAMP sensors Epac. The Epac protein is N- and C-terminally fused to two fluorophores. If cAMP is not bound to Epac, both fluorophores are in close proximity of less than 10 nm to each other resulting in a FRET signal. Binding of cAMP to Epac due to G_s_-coupled receptor activation causes conformational change of the protein which results in greater distance of both fluorophores which leads to FRET signal decreases. The *white circle* displays the cAMP binding site in the Epac protein; cAMP is displayed as red dot. **g** summary of FRET signal decreases induced by isoprenaline in the presence (*hatched bars*) or absence (*solid bars*) of the selective β_2_ receptor antagonist propranolol (1.5 mM). *Numbers* over *bars* indicate the numbers of measured cells and the number of individual coverslips from at least 3 experimental days. Significances tested between propranolol-treated and untreated cells. **P < 0.01, ***P < 0.001

To monitor variations of intracellular cAMP concentrations in living cells, biochemical approaches are frequently chosen. The classical method to detect receptor-mediated cAMP accumulation in intact cells is based on pre-labeling with ^3^H-adenine and subsequent calculation of the conversion to ^3^H-cAMP extracted from cell homogenates [24]. This radioactive, multi-cell-based method is highly sensitive and reproducible especially for monitoring G_s_-protein-induced cAMP increases. However, to analyze G_i/o_-protein-induced cAMP level decreases, physiologically low basal cAMP concentrations must be artificially increased by using the AC activator forskolin and/or the phosphodiesterase inhibitor IBMX. These measures might impact the validity and reliability of the assay and decrease the potency of G_i/o_-coupled receptor agonists. Another major drawback of the latter method is the lack of time-resolved and spatial assessment of cAMP fluctuations in single living cells. These limitations can be overcome by exploiting Förster resonance energy transfer (FRET) within exchange proteins directly activated by cAMP (Epac) that undergo a conformational change after cAMP binding [25]. This optical method is based on Epac1 or Epac2 proteins N- and C-terminally fused to two fluorophores, e.g., yellow fluorescent protein (YFP) and cyan fluorescent protein (CFP) with YFP representing the acceptor and CFP the donor of the fluorescence signal. Binding of cAMP to Epac results in a conformational change altering the distance between the two fluorophores and decreasing FRET signals [6, 17, 21] (for a schematic illustration, see Fig. 1a, inset). Until now, several Epac-based FRET sensors have been devised. These sensors are either based on Epac1 or Epac2 or on optimized Epac proteins. One of these optimized Epac proteins lacks the membrane-targeting DEP sequence (ΔDEP), and the catalytically active guanine nucleotide exchange factor (GEF) domain is disabled by a point mutation [21]. This cytoplasmic sensor detects cAMP changes in the physiological range from 0.1 to 100 μM [21] with improved FRET responses compared to Epac1- and Epac2-based sensors displaying EC_50_ values of 2.4 μM for YFP-Epac1-CFP and 0.9 μM for YFP-Epac2-CFP [17]. These sensors were additionally improved by insertion of different fluorophores: mTurquoise, a cyan fluorescent protein with more than doubled quantum efficiency, single-exponential fluorescence decay, and exceptional photo stability, was N-terminally fused to Epac [11], and an improved yellow fluorophore ^cp173^Venus-Venus was inserted at the C-terminus. The latter double acceptor consists of mVenus, a stable yellow fluorescent protein, and a circular permutation of Venus (^cp173^Venus) [16] resulting in enhanced brightness, acid stability, and stability to chloride changes. The combination of mTurquoise as a FRET donor and of ^cp173^mVenus-mVenus as a FRET acceptor resulted in higher FRET efficiencies, enhanced photo stability, and an increased dynamic range [11] and is referred to as the H74 construct [11]. Recently, additionally optimized Epac constructs were generated. The so-called H187 construct exhibits a 2.5-fold increased affinity to cAMP due to an additional amino acid exchange (Q270E) and displays higher photo stability and higher FRET efficiencies by means of combining the fluorophores mTurquoise2, the most photostable cyan fluorophore at present, and a tandem of ^cp173^Venus [10].

Although Epac-based cAMP sensors have constantly been improved over the last few years, the use of these sensors is largely restricted to the detection of cAMP increases mediated by G_s_-coupled receptors. Until now, Epac sensors have not been employed to monitor G_i/o_-mediated cAMP decreases without pre-stimulation with forskolin or IBMX to increase endogenous cAMP levels. To investigate whether FRET-based sensors can principally be exploited to reliably monitor cAMP level decreases following G_i/o_-activation and to systematically compare different Epac cAMP sensors, two novel Epac constructs with higher photo and acid stability based on the original Epac constructs Epac1 and Epac2 were generated by us. In addition, the original non-optimized [17] and the two abovementioned optimized FRET-based Epac sensors [10, 11] were tested as potential sensors of G_i/o_-coupled receptor activation. The systematic comparison of different Epac sensors allowed defining essential parameters governing the detection of cAMP level decreases in single cells.

## Results

### Optimized FRET-based cAMP sensors are effective in monitoring G_s_-mediated cAMP level increases

To investigate whether Epac-based cAMP sensors can be employed to detect cAMP decreases, we used a set of different Epac sensors: four sensors which are well-established to monitor G_s_-protein-mediated cAMP increases (YFP-Epac1-CFP, YFP-Epac2-CFP, H74, and H187) and two constructs with modified fluorochromes generated by us (mV-Epac1-mTq2, mV-Epac2-mTq2) which are based on the original Epac1 and Epac2 constructs. To test if all Epac-based cAMP constructs were functional, we performed FRET measurements with HEK293 cells endogenously expressing G_s_-coupled adrenergic β_2_-adrenergic receptors (β_2_Rs) that were transfected with one of the Epac constructs. Schematic structures of the five Epac sensors are displayed in Fig. 1a–f (left panels). Agonist stimulations with the β receptor agonist isoprenaline (200 μM) increased cyan and simultaneously decreased yellow fluorescence resulting in FRET signal decreases (Fig. 1a–f) that reflect elevations of cAMP concentrations. Our results show that all constructs were functional and suitable to detect cAMP increases. However, the amplitudes of FRET signals varied between −5.7 ± 0.7% in the case of mV-Epac1-mTq2 and −97.2 ± 12.9% in the case of the optimized Epac construct H187. The summary of FRET signal amplitudes shows that the FRET pair YFP/CFP was more efficacious than the FRET pair mVenus/mTurquoise2 (Fig. 1g). Interestingly, there were no significant differences in the detection of maximal cAMP increases between Epac1 and Epac2 constructs. The smallest FRET changes were obtained by the Epac1 and Epac2 constructs with the fluorophores mVenus and mTurqouise2 indicating that enhanced stability of fluorochromes does not necessarily result in higher FRET efficiency. Notably, highest FRET amplitudes were monitored using the cytosolic FRET constructs H74 (−43.8 ± 5.4%) and H187 that exhibited even 2.2-fold higher FRET amplitudes. These findings suggest that H74 and H187 constructs are most suitable to detect increases in cAMP concentrations concordant with observations by Klarenbeek et al. [10, 11].

### FRET-based cAMP sensors are suitable to monitor cAMP decreases in living cells induced by activation of G_i/o_-coupled α_2A_ receptors

**Fig. 2:**
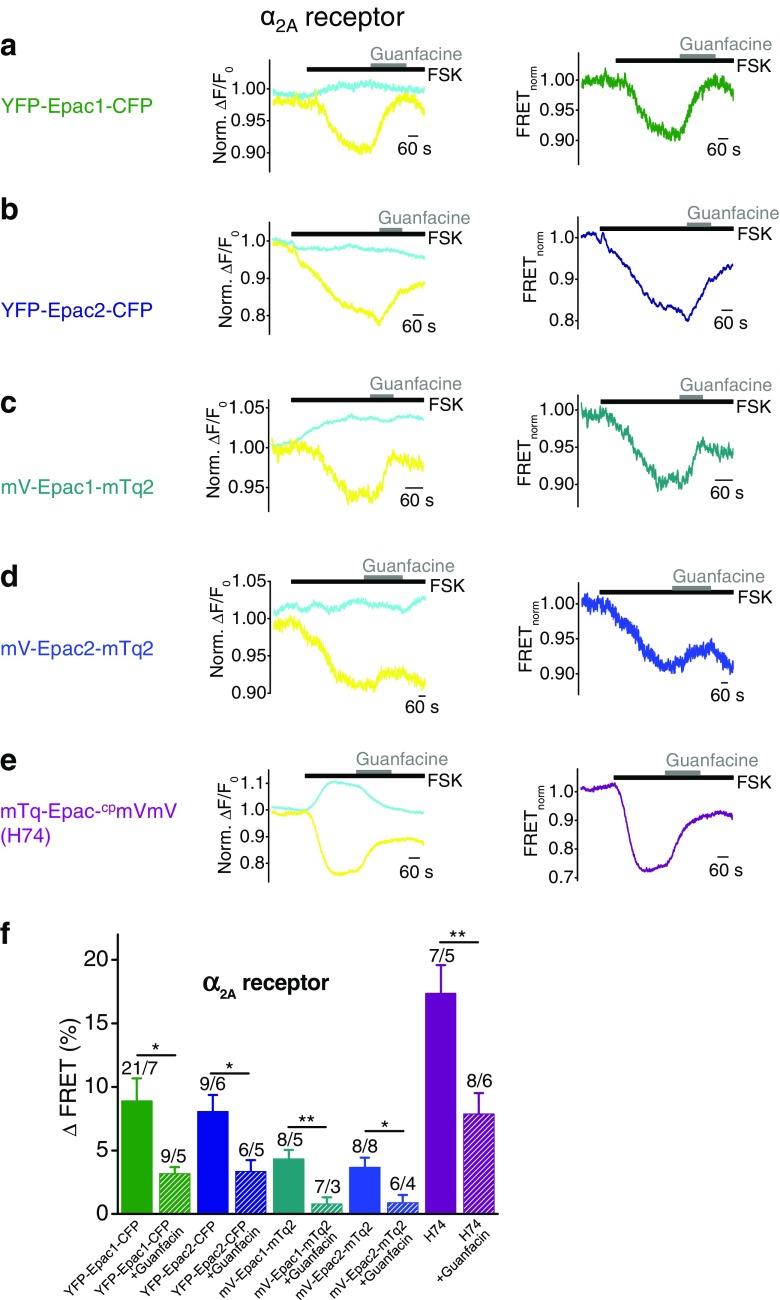
FRET-based cAMP sensors are suitable to monitor cAMP decreases in living cells induced by activation of G_i/o_-coupled α_2A_ receptors. FRET measurements with HEK293 cells expressing G_i/o_-protein-coupled α_2A_ receptors together with one of the indicated FRET-based cAMP sensors. **a**–**e** Representative FRET measurements are displayed showing time courses of the normalized yellow and cyan fluorescence signals (*left*) and of the normalized FRET signal (*right*). *Black bars* indicate application of the adenylyl cyclase activator forskolin (FSK) in submaximal concentration (1 μM) to increase basal cAMP levels. *Gray bars* show application of the selective α_2A_ receptor agonist guanfacine (250 μM). **f** Summary of FRET signal increases induced by guanfacine in the presence (*hatched bars*) or absence (*solid bars*) of the selective α_2A_ receptor antagonist yohimbine (1 mM). *Numbers* over *bars* indicate the numbers of measured cells and the number of individual coverslips from at least 3 experimental days. Significances tested between cells treated and not treated with yohimbine. *P < 0.05, **P < 0.01

To elucidate, whether Epac sensors are principally suitable to detect intracellular cAMP level decreases, we co-expressed G_i/o_-coupled α_2A_ receptors (α_2A_R) with the Epac constructs YFP-Epac1-CFP, YFP-Epac2-CFP, mV-Epac1-mTq2, and mV-Epac1-mTq2 and with the optimized sensor H74. Agonist stimulations with the selective α_2A_R agonist guanfacine (250 μM) were not sufficient to reliably increase FRET signals. Thus, we pre-treated cells with forskolin, an activator of AC, at a submaximal concentration (1 μM) to slightly increase basal cAMP levels. Higher concentrations of forskolin up to 10 μM caused maximal cAMP accumulation that exceeded the dynamic range and could therefore not be reversed by application of agonists. Pre-incubation with 1 μM forskolin *plus* 10 μM IBMX was not more effective than forskolin alone (data not shown) and pre-incubation of 100 μM 8-Bromo-cAMP did not result in any reliable measurements (data not shown). Thus, employing these Epac constructs without forskolin application, a reduction of intracellular cAMP concentrations could not be reliably determined, similar to the situation with radioactive cAMP accumulation assays. However, as soon as stable fluorescence baselines were reached, application of 1 μM forskolin entailed increases of cyan and decreases of yellow fluorescence resulting in decreased FRET signals (Fig. 2a–e). When stable fluorescence baselines were established again, the selective agonist guanfacine was applied resulting in FRET signal increases (Fig. 2a–e) reflecting decreases of intracellular cAMP concentrations. The summary of FRET signal amplitudes illustrates that the H74 construct was most efficient to detect cAMP decreases with FRET signal amplitudes of 17.4 ± 2.2% (Fig. 2f). Just as in the case of G_s_-dependent cAMP accumulation, the FRET pair YFP/CFP resulted in FRET signals higher than the mVenus/mTurquoise2 FRET pair, and there were no significant differences in the maximal cAMP decreases between Epac1 and Epac2 constructs. The smallest changes of FRET signals of 3.7 ± 0.8% were monitored when using mVenus-Epac2-mTurquoise2. Altogether, these findings suggest that Epac-based cAMP sensors are suitable tools to monitor G_i/o_-protein-mediated decreases of intracellular cAMP concentrations in single living cells if cells are pre-treated with forskolin to elevate basal cAMP levels. In addition, these findings confirm that the FRET sensor H74 is highly effective in detecting cAMP level fluctuations.

### FRET-based cAMP sensors reliably detect cAMP level decreases induced by activation of G_i/o_-protein-coupled μ opioid receptors

**Fig. 3:**
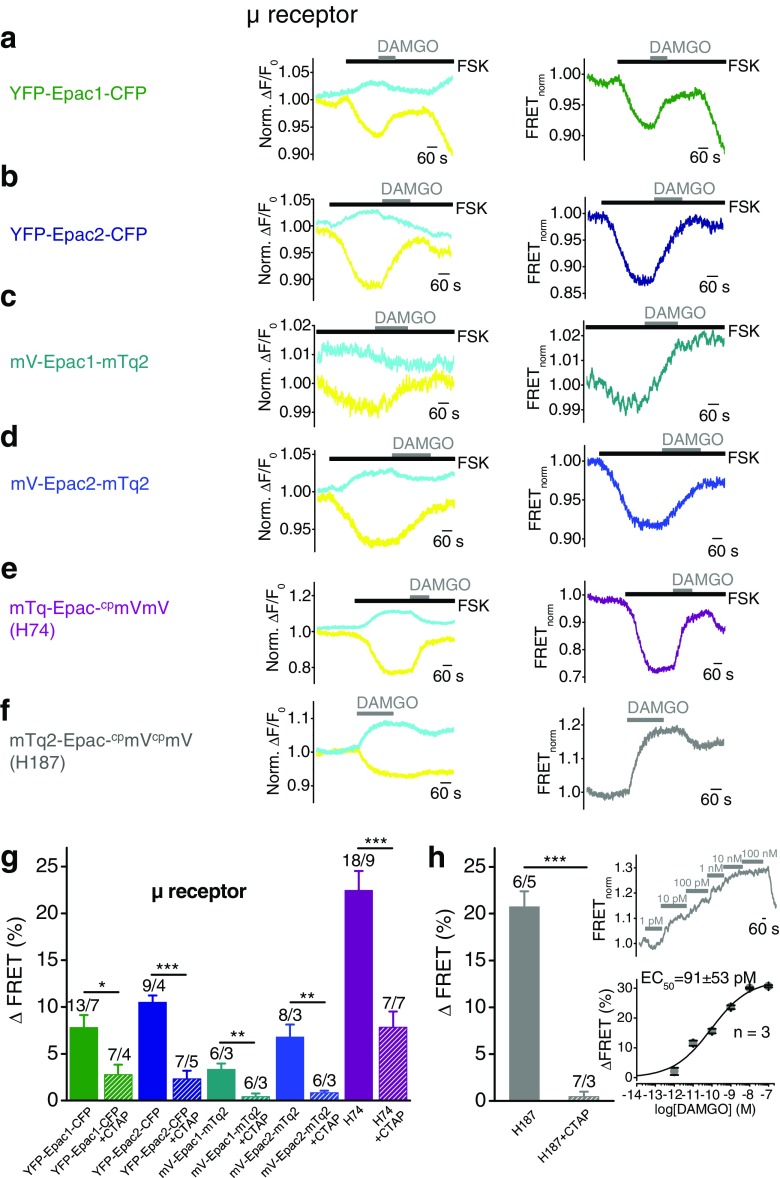
FRET-based cAMP sensors reliably detect cAMP level decreases induced by activation of G_i/o_-protein-coupled μ opioid receptors. FRET measurements with HEK293 cells expressing G_i/o_-protein-coupled μ opioid receptors together with one of the indicated FRET-based Epac constructs. **a**–**f** Representative FRET measurements are displayed showing time courses of the normalized yellow and cyan fluorescence signals (*left*) and of the normalized FRET signal (*right*). *Black bars* indicate application of the adenylyl cyclase activator forskolin (1 μM, FSK) in submaximal concentration to increase basal cAMP levels (**a**–**e**). *Gray bars* show application of the selective μ receptor agonist DAMGO (100 nM). **g** Summary of FRET signal increases induced by DAMGO after forskolin pre-stimulation in the presence (*hatched bars*) or absence (*solid bars*) of the selective μ receptor antagonist CTAP (500 nM). *Numbers* over *bars* indicate the numbers of measured cells and the number of individual coverslips from at least 3 experimental days. **h** Summary of FRET signal increases induced by 100 nM DAMGO in the presence (*hatched bars*) or absence (*solid bars*) of CTAP (500 nM). *Right insets* show representative FRET signal trace with application of increasing concentrations of DAMGO (*top*) and the concentration response curve displayed as mean ± s.e.m. of three independent measurements (*bottom*). The *curve* was fitted using the Hill equation. *Numbers* over *bars* indicate the numbers of measured cells and the number of individual cover slips from at least 3 experimental days. Significances tested between cells treated and not treated with CTAP. *P < 0.05, **P < 0.01, ***P < 0.001

Next, we tested μ opioid receptors (μRs) as another example of G_i/o_-coupled receptors. First, HEK293 cells co-expressing μRs together with one of the Epac sensors YFP-Epac1-CFP, YFP-Epac2-CFP, mV-Epac1-mTq, mV-Epac1-mTq, or H74 were investigated. One micromolar of forskolin had to be applied to slightly enhance basal cAMP concentrations similar to α_2A_R-expressing cells, since without this measure no significant FRET signal changes upon agonist application could be detected. Forskolin decreased FRET signals and subsequent application of the selective μR agonist DAMGO (100 nM), a synthetic opioid peptide, lead to an increase in FRET signals (Fig. 3a–e). The summary of differential FRET amplitudes shows that the H74 construct was most efficient in detecting cAMP decreases with FRET amplitudes of 22.5 ± 2.1% (Fig. 3f). Moreover, the FRET pair YFP/CFP was more effective than the FRET pair mVenus/mTurquoise2 and there were no significant differences in the maximal cAMP decreases between Epac1 and Epac2 constructs similar to the findings observed with α_2A_ and β_2_ receptors. These findings confirm that after pre-incubation with forskolin, G_i/o_-induced cAMP decreases can be effectively monitored by using the FRET technique.

Next, we asked whether the FRET sensor H187 characterized by a higher affinity to cAMP and a considerably increased dynamic range might be sensitive enough to detect a decline of cAMP levels without pre-treatment with forskolin. Indeed, analyzing HEK293 cells co-expressing μR and the H187 sensor, we found that agonist stimulation with DAMGO resulted in FRET signal increases (Fig. 3f) indicating that this sensor is suitable to monitor cAMP decreases without pre-incubation with forskolin. Maximal FRET signal increases were already detectable following application of 100 nM DAMGO. Higher concentrations did not cause higher FRET signal changes. The summary of FRET amplitudes shows that agonist stimulation with DAMGO evokes enhanced FRET signals of 20.7 ± 1.6% (Fig. 3h) similar to the FRET signals determined by analyzing the H74 construct in the presence of forskolin. The EC_50_ value for DAMGO determined by analyzing H187 and μRs co-expressing HEK293 cells was 91 ± 53 pM (*n* = 3) (Fig. 3h, inset) demonstrating a high potency of DAMGO. Altogether, these findings suggest that the H187 construct is a preferable molecular tool to monitor G_i/o_-protein-mediated decreases of cAMP levels in a concentration-dependent, time-resolved manner in single living cells without the need of forskolin pre-treatment.

### Agonist-induced FRET signal changes can be suppressed by selective receptor antagonists

**Fig. 4:**
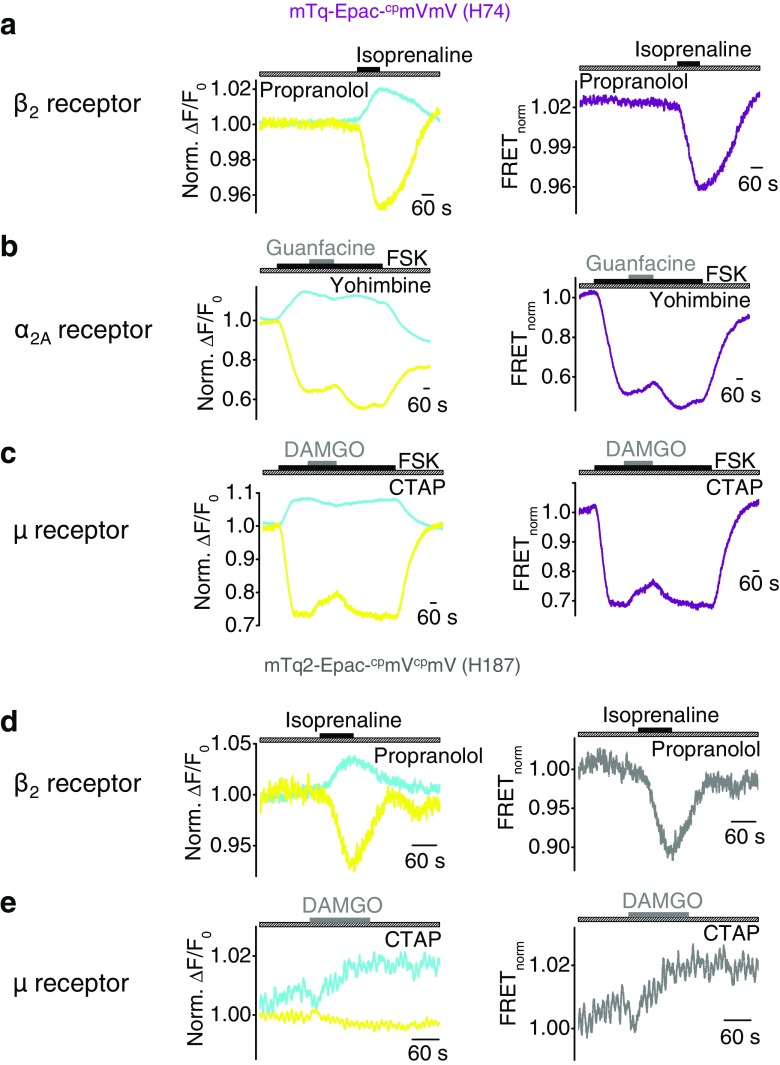
Agonist-induced FRET signal changes can be suppressed by selective receptor antagonists. FRET measurements with HEK293 cells endogenously expressing G_s_-protein-coupled β_2_Rs or over-expressing G_i/o_-protein-coupled α_2A_ or μ receptors together with the FRET-based Epac sensor H74 (**a**–**c**) or H187 (**d**, **e**). **a**–**e** Representative FRET measurements are displayed with time courses of the normalized yellow and cyan fluorescence signals (*left*) and of the normalized FRET signals (*right*). *Hatched bars* indicate application of the selective antagonists propranolol (1.5 mM, **a**, **d**), yohimbine (1.0 mM, **b**), and CTAP (500 nM, **c**, **e**). *Gray bars* show application of the agonists isoprenaline (200 μM), guanfacine (200 μM), and DAMGO (100 nM). **b**, **c** Application of the adenylyl cyclase activator forskolin (1 μM, FSK) in submaximal concentration to increase basal cAMP levels is displayed

To investigate whether agonist-induced FRET signals corresponding to increases or decreases of cAMP levels are specific, selective receptor antagonists were applied. We used propranolol (1.5 mM) to block endogenously expressed β_2_Rs (Fig. 4a, d), yohimbine (1 mM) to block α_2A_Rs (Fig. 4b), and the synthetic cyclic penicillamine-containing octapeptide (CTAP) (500 nM) to block μRs (Fig. 4c, e). Antagonists were applied at concentrations that precluded receptor activation in the presence of maximal agonist concentrations. Applying the β_2_R antagonist propranolol, FRET signals were remarkably smaller compared to untreated cells (Figs. 1g and 4a, d). Similar results were observed analyzing α_2A_R or μR and H74 or H187 sensor co-expressing cells with or without pre-stimulation with forskolin in the presence of yohimbine or CTAP (Figs. 2f, 3g, h, and 4b, c, e). Summarized FRET signal changes upon application of selective antagonists show that antagonists significantly reduce agonist-induced FRET signals elicited by G_s_- and G_i/o_-coupled receptors (Figs. 1g, 2f, and 3g, h) indicating that the observed FRET responses are specific. However, FRET signals were not completely abrogated by receptor antagonists.

### Prerequisites for reliable and reproducible measurements with FRET-based Epac sensors to detect G_i/o_-protein-mediated cAMP decreases

**Fig. 5:**
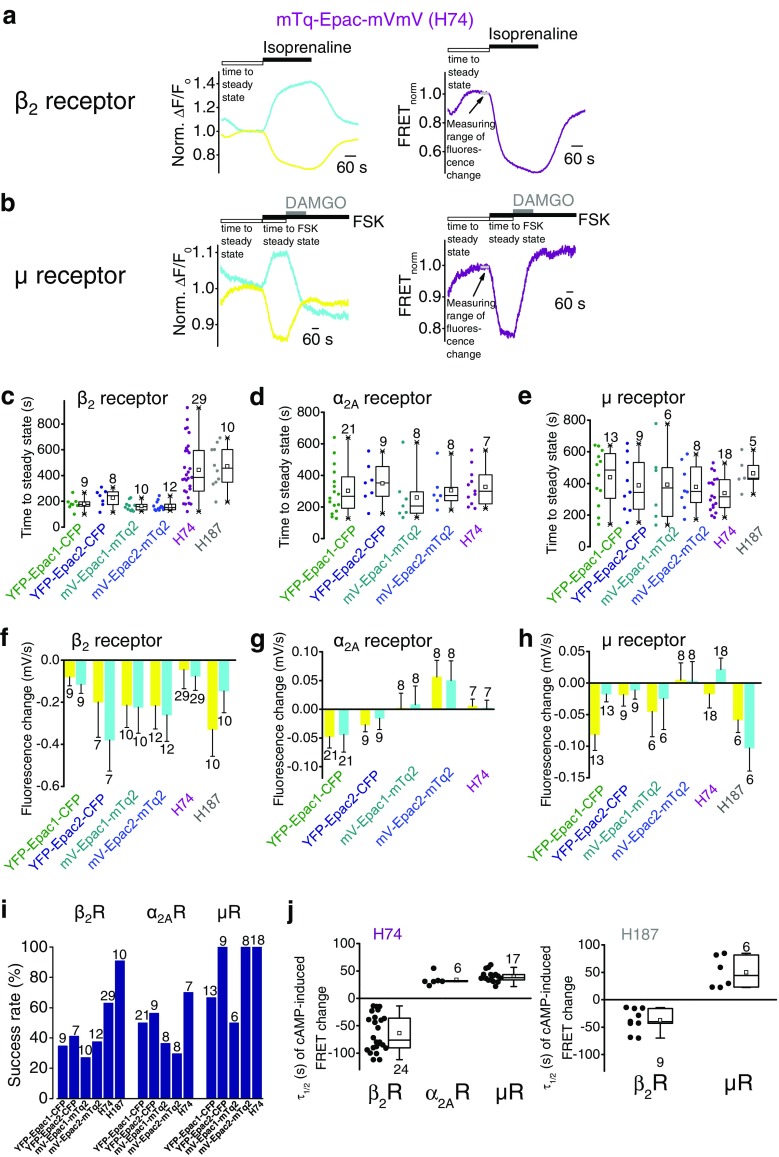
Prerequisites for reliable and reproducible measurements with FRET-based Epac sensors to detect G_i/o_-protein-mediated cAMP decreases. For reliable and reproducible FRET measurements with FRET-based Epac sensors, achievement of steady-state conditions prior to application of different stimuli was of utmost importance. **a**, **b** Representative FRET measurement of HEK293 cells endogenously expressing β_2_Rs (**a**) or over-expressing μRs (**b**) and the H74 construct. The time courses of the normalized yellow and cyan fluorescence signals (*left*) and of the normalized FRET signal (*right*) are displayed. Isoprenaline (200 μM, *black bar*, **a**) or forskolin (1 μM, FSK, *black bar*, **b**) are applied at steady-state conditions (time to steady state is indicated as *white bar*). The second stimulus DAMGO (100 nM, *gray bar*) is applied when steady-state conditions are achieved (time to steady state of FSK is indicated as a white bar, **b**). **a**, **b** *Light gray bars* indicate steady-state conditions used to determine the range of fluorescence changes as millivolts per second (*left*). **c**–**e** Summaries of the times to steady state of β_2_R (**c**), α_2A_R (**d**), or μR (**e**) and different indicated Epac sensor-expressing HEK293 cells. Results are displayed as boxplot analysis plus single values. *Squares* indicate mean values. *Numbers* over *bars* indicate the numbers of measured cells from at least 3 experimental days. **f**–**h** Slopes of the yellow and cyan florescence traces of β_2_R (**f**), α_2A_R (**g**), or μR (**h**) and different Epac sensor-expressing HEK293 cells determined before application of the first stimulus. *Fluorescence was detected as voltage of the transimpedance amplifier from the photodiode. **i** Summary of success rates of FRET measurements with HEK293 cells co-expressing different receptors and indicated FRET-based Epac sensors. *Numbers* over *bars* indicate the numbers of measured cells from at least 3 experimental days. **j**, **k** Analysis of the kinetics of FRET signal changes using the H74 (**j**) or the H187 sensor (**k**) after G_s_- or G_i/o_-coupled receptor activation calculated as exponential time constant as τ_1/2_ displayed as boxplot analysis plus single values. *Squares* indicate mean values. *Numbers* indicate the numbers of measured cells from at least 3 experimental days

To obtain reliable and reproducible results using FRET-based Epac sensors to detect G_i/o_-protein-mediated cAMP decreases with or without pre-treatment with forskolin, it is of paramount importance to equilibrate the system and reach steady-state conditions before applying any stimuli. Representative FRET measurements of HEK293 cells endogenously expressing β_2_Rs or transiently over-expressing μRs which were additionally transfected with H74 are displayed in Fig. 5a, b to illustrate the time period to steady-state conditions and the measuring range of the fluorescence decay at steady-state conditions that must be established before application of the first stimulus. Steady-state conditions are characterized by constant fluorescence traces without apparent fluorescence decay of both fluorescence traces. In some experiments, after application of forskolin (Fig. 5b), we had to wait for steady-state equilibration a second time before applying DAMGO entailing FRET signal increases. The times to steady state before applying the first stimulus varied between Epac constructs and the analyzed receptors from 100 to 926 s with a mean time of 345 ± 13 s (Fig. 5c–e). The second stimulus could be applied after a lag period of 390 ± 17 s on average. Summarized fluorescence changes of cyan and yellow fluorescence signals determined as millivolts per second before application of the first stimulus are displayed in Fig. 5f–h. Highest variations of fluorescence changes were found when analyzing HEK293 cells endogenously expressing β_2_Rs (Fig. 5f). Under these conditions, success rates of FRET measurements with different receptors using the indicated cAMP sensors were as follows (Fig. 5i): Analyzing HEK293 cells endogenously expressing β_2_Rs or over-expressing α_2A_Rs, we observed that 27 to 56% of all measurements were successful employing the non-optimized Epac1- and Epac2-based FRET sensors. The optimized H74 construct yielded success rates ranging between 61 and 70%. The H187 construct showed a success rate of 91% analyzing endogenously expressed β_2_Rs. Moreover, 53% of measurements showed FRET signal increases induced by agonist stimulation in the case of μRs co-expressing cells without forskolin pre-treatment. Interestingly, the success rates not only depended on the Epac construct but also on the type of receptor. Measurements with the μR after forskolin pre-treatment were most successful with success rates up to 100%. There were no differences between success rates using the optimized H74 construct or YFP-Epac2-CFP or mV-Epac2-mTq2. In these experiments, the Epac2 sensors and H74 performed equally well. However, the H74 sensor is characterized by a wider dynamic range with higher FRET amplitudes rendering H74 the most preferable cAMP sensor. Although the success rate using the H187 construct with μRs co-expressing cells was lower, this construct showed an adequate dynamic range (about 20% FRET signal increase) and had an increased affinity to cAMP. Thus, this construct is well suitable to monitor small changes of intracellular cAMP concentrations without artificially increasing basal cAMP levels.

To analyze the kinetics of FRET signal changes after receptor stimulation, we fitted the FRET signal curves of H74 and H187 sensor-expressing cells during agonist stimulation by a monoexponential function. The kinetics of α_2A_R- and μR-induced FRET signal changes were not significantly different (Fig. 5j, k) independent of the FRET sensor employed. Notably, the kinetics of cAMP increases by stimulation of endogenous β_2_Rs using H74 and H187 constructs was not significantly different and showed absolute values of time constants (half-time, τ_1/2_) similar to those observed with G_i/o_-coupled receptors (Fig. 5j, k). These findings support the conclusion that the cAMP sensors H74 and H187 are suitable to determine the kinetics not only of cAMP level increases by G_s_-activation but also of cAMP decreases subsequent to G_i/o_-stimulation.

## Discussion

Dynamic intramolecular FRET using Epac-based cAMP sensors to determine intracellular cAMP concentration variations resulting from activation of G_s_-coupled receptors in living cells is a well-established method. Until now, this technique was applied to monitor G_s_-induced cAMP level increases. In this study, we optimize the methodology and show for the first time that Epac-based cAMP sensors are sufficiently dynamic to allow for the detection of G_i/o_-dependent cAMP decreases as well. Interestingly, employing the most sensitive Epac sensor H187, we found that artificially increasing endogenous basal cAMP levels with forskolin or IBMX was not necessary to monitor G_i/o_ effects. Forskolin and IBMX are widely used to increase basal cAMP levels in order to monitor cAMP level decreases mediated by G_i/o_-protein activation. The adenylyl cyclase activator forskolin leads to cAMP increases resulting in protein kinase A (PKA) activation thereby changing the overall phosphorylation status, which might cause sensitization or desensitization of GPCRs and other signaling proteins. IBMX is a non-selective phosphodiesterase inhibitor which additionally increases cyclic guanosine monophosphate (cGMP) levels. Moreover, IBMX inhibits tumor necrosis factor TNFα [5, 14] and is a non-selective adenosine receptor antagonist [26]. Altogether, IBMX and forskolin engage several signaling pathways thereby evoking adverse effects, which might influence the receptor status. Thus, avoiding the use of these substances is a major advantage.

In our study, G_i/o_-protein-mediated FRET signal increases were receptor-specific and could be significantly suppressed by selective antagonists similar to G_s_-protein-mediated FRET signals. Using FRET sensors based on Epac1 and Epac2 and the optimized H74 construct, we found that a prerequisite for reliable FRET measurements of G_i/o_-mediated cAMP decreases was pre-treatment of the cells with the adenylyl cyclase activator forskolin at submaximal concentrations similar to biochemical multi-cell radioactive labeling assays. Since these sensors were well suitable for the detection of FRET increases corresponding to cAMP decreases induced by α_2A_ and μ receptors, these findings show that Epac sensors can be employed to analyze G_i/o_-activation. However, quality and reproducibility of measurements strongly depended on the establishment of steady-state conditions of the fluorescence signal prior to application of forskolin and/or agonists. There were differences between the different FRET constructs regarding the success rates and the dynamic range of the observed FRET signals. The H74 construct was found to be preferable when monitoring FRET signals induced by G_i/o_-proteins subsequent to forskolin pre-treatment. However, forskolin administration was no longer required when using the latest FRET construct H187 which exhibits a higher affinity to cAMP. This sensor showed a higher dynamic range than the H74 sensor analyzing G_s_-coupled receptor activation. Moreover, due to enhanced cAMP affinity, the H187 sensor already responds to small cAMP alterations. Therefore, the latter sensor allowed for the detection of decreased basal cAMP levels in HEK293 cells obviating the need for pre-stimulation with forskolin. Interestingly, using the H187 construct, the EC_50_ value for DAMGO was decreased to 91 pM indicating a superior sensitivity. Employing the classical radioactive assay based on the conversion to ^3^H-cAMP, a nearly 100-fold higher EC_50_ value of 8.4 nM was determined for DAMGO [4]. Notably, a comparable EC_50_ value of 320 pM was calculated when monitoring DAMGO-induced calcium transients in CHO-K1 cells stably expressing μRs and Gα_15_ proteins [8] without forskolin pre-incubation. Thus, circumventing pre-stimulation with forskolin appears to increase the potency of agonists and the overall sensitivity of the assay. Moreover, we monitored FRET signals on the single cell level instead of performing multicellular assays in which the signal represents an average of all cells including non-responding and damaged cells that may affect sensitivity. Thus, taking advantage of the H187 sensor represents a considerable improvement compared to multicellular radioactive assays.

Another prerequisite for reliable measurements of G_i/o_-mediated cAMP level decreases consists of equilibration of the system to achieve steady-state conditions of fluorescence signals prior to stimulation. Under these conditions, FRET measurements were characterized by success rates of 27 to 100% depending on the FRET construct and the receptor analyzed. Although all Epac sensors can principally be applied to dynamically monitor intracellular cAMP concentrations, the dynamic ranges of FRET signals varied considerably. Constructs mV-Epac1-mTq2 and mV-Epac2-mTq2 engineered by us showed the smallest FRET signal amplitudes, while H74 and H187 constructs had the highest dynamic range.

Apart from Epac sensors, there are several other methods, which are suitable to measure G_i/o_-protein-mediated signaling. Generally, they can be classified as multi-cell and single-cell assays. Multi-cell assays comprise radioactive and non-radioactive methods and can either be based on the analysis of cell membrane fractions or of intact living cells. Notably, analysis of membrane preparations can only give information at one time point and is not suitable to detect dynamic changes of cellular signals. A common approach to monitor G-protein activation using cell membrane preparations is the radioactive ^[35S]^GTPγS binding assay which is highly sensitive [15] and does not require pre-stimulation with forskolin. However, ^[35S]^GTPγS binding studies are not selective for G_i/o_-protein activation. Other multi-cell assays monitor cAMP accumulation as an end point measurement without time-resolved monitoring of cAMP changes. In addition to the classical ^3^H-adenine pre-labeling approach, competition assays based on radiometric or immunoassay techniques are widely used. Several radiometric cAMP accumulation assays such as RIA, ELISA, scintillation, and chemiluminescence proximity assays are commercially available and are even suitable for high-throughput screening (summarized in [9]). Other multi-cell approaches use label-free technologies with electrical or optical measurement systems (summarized in [7, 32]) and can monitor GPCR activation in intact living cells. Interestingly, these approaches allow for real-time detection of signal changes. However, the signal is only a summation of all biochemical, physiological, and morphological responses of cells and neither provides detailed information about the signaling cascade nor does it allow to draw conclusions about cAMP levels. Altogether, multi-cell assays are not suitable to detect cAMP changes with spacial and temporal resolution.

On the single cell level, G_i/o_-protein activation can be monitored with various biosensors. One possibility is the determination of G-protein activation in single living cells using biosensors based on the dissociation of the heterotrimer after G-protein activation. An example is the detection of G_i/o_-activation employing the FRET technique which comprises the use of Gα_i_ FRET sensors [30]. This method is based on intermolecular FRET between ^cp173^mVenus-Gγ as a FRET acceptor and Gα_i_-mTurquoise2 as a FRET donor. These sensors allow the recording of the fast kinetics of G_i_-activation. However, the method relies on the over-expression of G-protein subunits which may strongly influence the stoichiometry of receptors and G-proteins thereby impacting activation kinetics and potentially obfuscating the physiological preference of a given receptor for a defined composition of heterotrimeric G-proteins. FRET measurements with the Epac sensors benefit from the fixed 1:1 or 1:2 donor/acceptor stoichiometry which cannot be provided by using a multicistronic expression vector for three G-protein subunits. In the case of using the Epac sensors, endogenous G-proteins are not influenced. Thus, Epac sensors may be molecular tools of choice if intracellular cAMP concentrations are to be monitored as a readout for G_i/o_-activation. Conversely, Gα_i_ FRET sensors may be advantageous when intending to characterize the kinetics of G_i_-activation.

Apart from Epac sensors, cAMP-binding biosensors are available which are based on protein kinase A (PKA) or on cyclic nucleotide-gated (CNG) channels. PKA-based sensors consist of catalytic and regulatory subunits of PKA, which are labeled with fluorophores. In the absence of cAMP, the subunits form a tetrameric holoenzyme complex. Binding of four cAMP molecules to the regulatory subunits causes dissociation of the catalytic subunits resulting in FRET signal decreases (summarized in [9]). Interestingly, PKA- and Epac-based biosensors can be targeted to the plasma membrane resulting in more rapid signals with greater amplitudes. In addition, mitochondria- and nuclear-targeted cAMP FRET sensors have been developed [6] which allow for subcellular analysis of cAMP signals. However, a disadvantage of PKA biosensors is their slower kinetics compared to Epac sensors. CNG and related hyperpolarization-activated cyclic nucleotide-gated (HCN) channels can also be used as cAMP biosensors. These biosensors were optimized to obtain a selectivity for cAMP over cGMP. cAMP signal increases cause channel activation which can be measured by performing patch-clamp measurements or calcium imaging (summarized in [19, 27, 31]). The major advantage of this biosensor is the temporal resolution of the cAMP signal rendering this sensor well suitable for kinetic analysis. In addition, a cytosolic HCN channel-based FRET biosensor (HCN2-camps) was generated which even allows for analysis of cAMP changes in subcellular compartments [18]. All CNG channel-based biosensors are well suitable to monitor cAMP level increases. However, these biosensors require high basal cAMP levels to detect G_i/o_-mediated cAMP level decreases and are therefore not optimal for the analysis of G_i/o_-mediated signaling. An alternative approach to measure G_i/o_-mediated signaling is the analysis of G-protein-gated inwardly rectifying potassium channels Kir 3.1–3.4 [1] which are directly activated by βγ subunits of G_i/o_-proteins [13]. However, direct monitoring of cAMP levels is not possible. Furthermore, the use of the patch-clamp technique as a readout is time-consuming and technically demanding.

Altogether, our findings provide a robust experimental framework allowing to utilize Epac-based cAMP sensors to functionally characterize G_i/o_-coupled receptors and to monitor cAMP decreases upon G_i/o_-activation in single living cells under physiological conditions.

## Methods

### FRET-based cAMP sensors used in the study

The FRET-based cAMP sensors eYFP-hEpac1-eCFP (YFP-Epac1-CFP) and eYFP-mEpac2B-eCFP (YFP-Epac2-CFP) in pcDNA3.1 vector were used [17]. To obtain more stable fluorochromes with higher quantum efficiency, photo stability, and strictly single-exponential fluorescence decay, we exchanged enhanced cyan fluorescent protein (eCFP) with mTurquoise2 and enhanced yellow fluorescent protein (eYFP) with the less pH- and Cl^−^-sensitive yellow fluorescent protein mVenus. For this, the inserts mVenus and mTurqoise2 additionally containing restriction sites for HindIII and EcoRI and for Xbal and NotI were amplified with by PCR using the following primer pairs: for mVenus 5′-AAA TTA AGC TTA TGG TGA GCA AGG GCG AGG A-3′ (sense) and 5′-AAA TTG AAT TCC TTG TAC AGC TCG TCC ATG C-3′ (anti sense) and for mTurquoise2 5′-AAA TTT CTA GAG TGA GCA AGG GCG AGG AGC T-3′ (sense) and 5′-AAA TTG CGG CCG CTT ACT TGT ACA GCT CGT CCA T-3′ (anti sense). The complementary DNA (cDNA) templates YFP-Epac1-CFP and YFP-Epac2-CFP in pcDNA3.1 vector were digested with XbaI und NotI to cut out eCFP. Next, eCFP was replaced by ligation with mTurquoise2. The mTurqoiuse2-containing cDNA templates were digested with HindIII und EcoRI to remove eYFP that was subsequently replaced by mVenus. Thus, we obtained two new FRET sensors: mVenus-hEpac1-mTurqouise2 (mV-Epac1-mTq2) and mVenus-mEpac2B-mTurquoise2 (mV-Epac2-mTq2). Moreover, the following optimized Epac-based constructs were used: mTurquoise-Epac-^cp173^mVenus-mVenus (mTq-Epac-^cp^mVmV or H74 [11]) which lacks the membrane-targeting DEP sequence (ΔDEP) and catalytic activity due to an amino acid exchange in the guanine nucleotide exchange factor (GEF) domain [21] and the construct mTurquoise2-Epac(Q270E)-^cp173^mVenus-^cp173^mVenus (mTq2-Epac-^cp^mV^cp^mV or H187 [10]) with an additional point mutation resulting in higher cAMP affinity.

### Cell culture and transfections

Human embryonic kidney (HEK293) cells were maintained in Earl’s minimal essential medium (Sigma-Aldrich, Taufkirchen, Germany) supplemented with 100 U ml^−1^ penicillin, 100 μg ml^−1^ streptomycin, 10% fetal calf serum (FCS, Gibco, USA), and 2 mM glutamine and held at 37 °C in a humidified atmosphere with 5% CO_2_. For FRET measurements, HEK293 cells were seeded into six-well plates and transfected with one of the following FRET-based cAMP sensors, 0.4 μg eYFP-Epac1-eCFP, 0.4 μg eYFP-Epac2-eCFP, 0.3 μg mVenus-Epac1-mTurquoise2, 0.3 μg mVenus-Epac2-mTurquoise2 or 0.3 μg mTurquoise-Epac-^cp173^mVenus-mVenus (H74 construct), and 0.3 μg mTurquoise2-Epac(Q270E)-^cp173^mVenus-^cp173^mVenus (H187 construct), and with one of the following GPCRs, 1 μg human α_2A_ adrenoceptor (NM_000681) or 1 μg human μ receptor (AY521028). For analysis of G_s_-activation, endogenously expressed β_2_ receptors were used. HEK293 cells were transfected at a cell confluency of about 90% by lipofection with GeneJuice® (Merck Millipore, Schwalbach, Germany) according to the manufacturer’s instructions. Cells were measured 24 h after transfection. Prior to FRET experiments, transfected HEK293 cells were seeded onto glass bottom dishes (FluoroDish Cell Culture Dish, 35 mm with glass bottom 23 mm, WPI, Berlin, Germany) coated with poly-l-lysine (Sigma-Aldrich). For coating, 1 ml poly-l-lysine solution (0.1 mg ml^−1^) was applied and incubated at room temperature for 60 min. After washing with 2 ml sterile Dulbecco’s phosphate-buffered saline (DPBS, Sigma-Aldrich), cells were seeded onto coated glass bottom dishes approximately 15 h prior to experimentation.

### FRET measurements

To measure changes of intracellular cAMP concentrations in single living cells mediated by G_s_- or G_i/o_-coupled receptor activation, FRET-based Epac sensors were used as described previously [17]. In brief, FRET experiments were carried out at room temperature and were conducted using a dual-emission photometry system (TILL Photonics, Planegg, Germany) on the stage of an Olympus IX70 inverted microscope equipped with an UPlanSAPO 100×/1.40 oil objective (Olympus, Hamburg, Germany). Upon excitation at 430 nm with Polychrome V (Till Photonics), fluorescence intensities at 480 ± 20 and 535 ± 15 nm were measured with the dual-emission photometry system using a beam splitter DCLP 505 nm. Emission was measured as voltage of the transimpedance amplifier of the photodiodes with a frequency of 5 kHz and was collected by an EPC10 amplifier (HEKA, Lambrecht, Germany) with the PATCHMASTER software (HEKA). FRET ratios were determined as ratios of eYFP, mVenus, ^cp173^mVenus-mVenus, or ^cp173^mVenus-^cp173^mVenus and eCFP, mTurquoise, or mTurquoise2 emissions. Normalized ratios were calculated from the corrected emission intensities. Fluorescence was corrected off-line for bleed-through of CFP (48%) or mTurquoise and mTurquoise2 (41.0%) into the 535 nm channel. Likewise, bleed-through of eYFP (6.3%), mVenus, ^cp173^mVenus-mVenus, or ^cp173^mVenus-^cp173^mVenus (6.3%) into the 480 nm channel was subtracted off-line. The corrected fluorescence was used to calculate the corrected FRET ratio. Fluorescence traces were not corrected for photo-bleaching since stimuli were only applied when constant fluorescence values and steady-state conditions were reached. During measurements, cells were continuously superfused with HEPES-buffered saline (HBS) solution containing 140 mM NaCl, 5.4 mM KCl, 1 mM MgCl_2_, 2 mM CaCl_2_, 10 mM glucose, and 10 mM HEPES (pH 7.4 with NaOH) resulting in an osmolarity of 295–302 mOsm kg^−1^. In some experiments, basal intracellular cAMP levels prior to G_i/o_-protein activation were increased by superfusion with HBS solution additionally containing submaximal concentrations of forskolin (1 μM, BIOZOL, Eching, Germany). Agonist stimulations were performed by applying guanfacine (250 μM, Tocris, Wiesbaden-Nordenstadt, Germany), isoprenaline (200 μM, Sigma-Aldrich), and DAMGO (100 nM, Sigma-Aldrich) in maximal effective concentrations. For some experiments, the selective receptor antagonists yohimbine (1 mM, Sigma-Aldrich), propranolol (1.5 mM, Sigma-Aldrich), or CTAP (500 nM, Tocris) were added to the bath solutions in concentrations that were effective to reverse agonist-induced cAMP level alterations.

### Statistical analysis

Data are presented as means ± standard error of the mean (s.e.m.). Unless stated otherwise, data were compared by a paired or unpaired Student’s *t* test, if a Gaussian distribution was confirmed by applying a Shapiro-Wilk (normality) test, and significance was accepted at *P* < 0.05 (*P < 0.05, **P < 0.01, ***P < 0.001, n.s. *P* > 0.05). Some pieces of data were displayed by boxplot analysis (percentiles 25 and 75%) with additional mean values. The changes of FRET signals during agonist stimulation were fitted with a mono-exponential function applying simplex algorithms and Levenberg-Marquardt iterations, until no reduction of chi-square was notable. Bi-exponential functions did not provide a better fit. Measurements were excluded if the fit did not converge. For calculation of EC_50_ values, a concentration response curve was fitted using the single Hill equation until no reduction of chi-square was notable.

## References

[1] Alexander SP, Catterall WA, Kelly E, Marrion N, Peters JA, Benson HE, Faccenda E, Pawson AJ, Sharman JL, Southan C, Davies JA, Collaborators C (2015). The Concise Guide to PHARMACOLOGY 2015/16: voltage-gated ion channels. Br J Pharmacol.

[2] Ben-Chaim Y, Chanda B, Dascal N, Bezanilla F, Parnas I, Parnas H (2006). Movement of ‘gating charge’ is coupled to ligand binding in a G-protein-coupled receptor. Nature.

[3] Birk A, Rinne A, Bunemann M (2015). Membrane potential controls the efficacy of catecholamine-induced beta1-adrenoceptor activity. J Biol Chem.

[4] Blake AD, Bot G, Freeman JC, Reisine T (1997). Differential opioid agonist regulation of the mouse mu opioid receptor. J Biol Chem.

[5] Deree J, Martins JO, Melbostad H, Loomis WH, Coimbra R (2008). Insights into the regulation of TNF-alpha production in human mononuclear cells: the effects of non-specific phosphodiesterase inhibition. Clinics.

[6] DiPilato LM, Cheng X, Zhang J (2004). Fluorescent indicators of cAMP and Epac activation reveal differential dynamics of cAMP signaling within discrete subcellular compartments. Proc Natl Acad Sci U S A.

[7] Fang Y, Frutos AG, Verklereen R (2008). Label-free cell-based assays for GPCR screening. Comb Chem High Throughput Screen.

[8] GenScript (2016) Human recombinant μ-opioid receptor OPRM1 stable cell line. GenScript. http://www.genscript.com/site2/document/11181_20091030025917.PDF.

[9] Hill SJ, Williams C, May LT (2010). Insights into GPCR pharmacology from the measurement of changes in intracellular cyclic AMP; advantages and pitfalls of differing methodologies. Br J Pharmacol.

[10] Klarenbeek J, Goedhart J, van Batenburg A, Groenewald D, Jalink K (2015). Fourth-generation epac-based FRET sensors for cAMP feature exceptional brightness, photostability and dynamic range: characterization of dedicated sensors for FLIM, for ratiometry and with high affinity. PLoS One.

[11] Klarenbeek JB, Goedhart J, Hink MA, Gadella TW, Jalink K (2011). A mTurquoise-based cAMP sensor for both FLIM and ratiometric read-out has improved dynamic range. PLoS One.

[12] Lefkimmiatis K, Zaccolo M (2014). cAMP signaling in subcellular compartments. Pharmacol Ther.

[13] Luscher C, Slesinger PA (2010). Emerging roles for G protein-gated inwardly rectifying potassium (GIRK) channels in health and disease. Nat Rev Neurosci.

[14] Marques LJ, Zheng L, Poulakis N, Guzman J, Costabel U (1999). Pentoxifylline inhibits TNF-alpha production from human alveolar macrophages. Am J Respir Crit Care Med.

[15] Milligan G (2003). Principles: extending the utility of [35S]GTP gamma S binding assays. Trends Pharmacol Sci.

[16] Nagai T, Yamada S, Tominaga T, Ichikawa M, Miyawaki A (2004). Expanded dynamic range of fluorescent indicators for Ca(2+) by circularly permuted yellow fluorescent proteins. Proc Natl Acad Sci U S A.

[17] Nikolaev VO, Bunemann M, Hein L, Hannawacker A, Lohse MJ (2004). Novel single chain cAMP sensors for receptor-induced signal propagation. J Biol Chem.

[18] Nikolaev VO, Bunemann M, Schmitteckert E, Lohse MJ, Engelhardt S (2006). Cyclic AMP imaging in adult cardiac myocytes reveals far-reaching beta1-adrenergic but locally confined beta2-adrenergic receptor-mediated signaling. Circ Res.

[19] Nikolaev VO, Lohse MJ (2006). Monitoring of cAMP synthesis and degradation in living cells. Physiology Bethesda, Md.

[20] Overington JP, Al-Lazikani B, Hopkins AL (2006). How many drug targets are there?. Nat Rev Drug Discov.

[21] Ponsioen B, Zhao J, Riedl J, Zwartkruis F, van der Krogt G, Zaccolo M, Moolenaar WH, Bos JL, Jalink K (2004). Detecting cAMP-induced Epac activation by fluorescence resonance energy transfer: Epac as a novel cAMP indicator. EMBO Rep.

[22] Rinne A, Birk A, Bunemann M (2013). Voltage regulates adrenergic receptor function. Proc Natl Acad Sci U S A.

[23] Rinne A, Mobarec JC, Mahaut-Smith M, Kolb P, Bunemann M (2015). The mode of agonist binding to a G protein-coupled receptor switches the effect that voltage changes have on signaling. Sci Signal.

[24] Salomon Y (1991). Cellular responsiveness to hormones and neurotransmitters: conversion of [3H]adenine to [3H]cAMP in cell monolayers, cell suspensions, and tissue slices. Methods Enzymol.

[25] Schmidt M, Dekker FJ, Maarsingh H (2013). Exchange protein directly activated by cAMP (epac): a multidomain cAMP mediator in the regulation of diverse biological functions. Pharmacol Rev.

[26] Snyder SH, Katims JJ, Annau Z, Bruns RF, Daly JW (1981). Adenosine receptors and behavioral actions of methylxanthines. Proc Natl Acad Sci U S A.

[27] Sprenger JU, Nikolaev VO (2013). Biophysical techniques for detection of cAMP and cGMP in living cells. Int J Mol Sci.

[28] Storch U, Mederos y Schnitzler M, Gudermann T (2012). G protein-mediated stretch reception. Am J Physiol Heart Circ Physiol.

[29] Strasser A, Wittmann HJ, Schneider EH, Seifert R (2015). Modulation of GPCRs by monovalent cations and anions. Naunyn Schmiedeberg’s Arch Pharmacol.

[30] van Unen J, Stumpf AD, Schmid B, Reinhard NR, Hordijk PL, Hoffmann C, Gadella TW, Goedhart J (2016). A new generation of FRET sensors for robust measurement of Galphai1, Galphai2 and Galphai3 activation kinetics in single cells. PLoS One.

[31] Willoughby D, Cooper DM (2008). Live-cell imaging of cAMP dynamics. Nat Methods.

[32] Zhang R, Xie X (2012). Tools for GPCR drug discovery. Acta Pharmacol Sin.

